# Covalent–Organic Framework-Based Materials in Theranostic Applications: Insights into Their Advantages and Challenges

**DOI:** 10.1021/acsomega.3c08456

**Published:** 2024-01-29

**Authors:** Harjot Kaur, Samarjeet Singh Siwal, Reena V. Saini, Vijay Kumar Thakur

**Affiliations:** † Department of Chemistry, M.M. Engineering College, Maharishi Markandeshwar (Deemed to be University), Mullana-Ambala, Haryana 133207, India; ‡ Biorefining and Advanced Materials Research Center, Scotland’s Rural College (SRUC), Kings Buildings, West Mains Road, Edinburgh EH9 3JG, U.K.; § Department of Biotechnology, MMEC, Maharishi Markandeshwar (Deemed to Be University), Mullana-Ambala, Haryana 133207, India

## Abstract

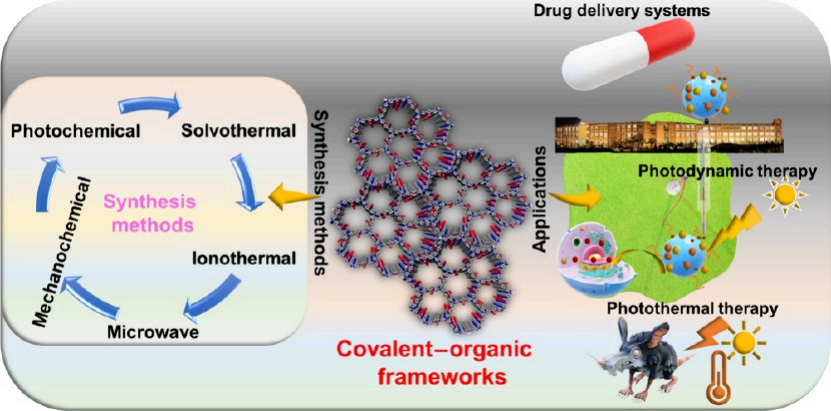

Nanomedicine has been essential in bioimaging and cancer therapy in recent years. Nanoscale covalent–organic frameworks (COFs) have been growing as an adequate classification of biomedical nanomaterials with practical application prospects because of their increased porosity, functionality, and biocompatibility. The high sponginess of COFs enables the incorporation of distinct imaging and therapeutic mechanisms with a better loading efficiency. Nevertheless, preliminary biocompatibility limits their possibility for clinical translation. Thus, cutting-edge nanomaterials with high biocompatibility and improved therapeutic efficiency are highly expected to fast-track the clinical translation of nanomedicines. The inherent effects of nanoscale COFs, such as proper size, modular pore geometry and porosity, and specific postsynthetic transformation through simple organic changes, make them particularly appealing for prospective nanomedicines. The organic building blocks of COFs may also be postmodified for particular binding to biomarkers. The exceptional features of COFs cause them to be an encouraging nanocarrier for bioimaging and therapeutic applications. In this review, we have systematically discussed the advances of COFs in the field of theranostics by providing essential features of COFs along with their synthetic methods. Further, the applications of COFs in the field of theranostics (such as drug delivery systems, photothermal, and photodynamic therapy) are discussed in detail with the help of available literature to date. Furthermore, the advantages of COFs over other materials for therapeutics and drug delivery are discussed. Finally, the review concludes with potential future COF applications in the theranostic field.

## 1 Introduction

Advanced porous materials have been designed, synthesized with specific structures, and investigated in different scientific fields.^1^ The advancement of porous material ranges from traditional inorganic materials (like silica, activated carbon, and zeolites) to organic–inorganic hybrid materials^2^ (like metal–organic frameworks (MOFs)^3^ and coordination polymers^4^). Among these porous materials, MOFs are produced via the self-assembly of metal ions and organic ligands through coordination linkages. MOFs and other materials have been widely utilized in the biomedical field and have also entered the stage of clinical trials.^5^ In 2005, a new generation of porous crystalline materials emerged as covalent–organic frameworks (COFs). COFs are a natural extension of MOFs and have been booming in recent years.^6^ COFs are 2D or 3D porous and crystalline materials formed by robust covalent interactions between organic precursors.

Recent advancements in COFs provide promising platforms that benefit therapeutic applications. COFs exhibit well-organized and long-range structure, and organic building blocks can be controlled positionally in two or three dimensions compared to amorphous organic polymers.^7^ These controlled structural properties can provide regular pores with large pore sizes, resulting in enhanced encapsulation of the theranostic agents. COFs can be synthesized facilely under mild conditions, and bonding defects may be involved at the boundary of the COF matrix.^8^ High porosity, internal channels and pores, and structural periodicity are advantageous in achieving high payloads and simplistic transport of therapeutic agents.^9^ The multifunctionality can be integrated into COFs by choosing suitable functionalized organic building blocks. This versatility of the COFs permits them to release and deliver theranostic agents or drugs in a precise way. In addition to this, robust covalent bonding present in COFs enables high chemical stability and biocompatibility. Recent reports confirmed that COFs could efficiently be utilized in theranostics with decent efficiency, performance, and lower systemic toxicity.^10^ The traction of COFs in the biomedical field is burgeoning and opens new paths for advancement in theranostic applications.^11^ However, more research is needed on the potential value of COFs in tissue engineering and regenerative medicine (TERM) to achieve a complete understanding of treatment and diagnosis by COFs in clinical translation.

Several reviews have explored the potential of COF-based materials in theranostic applications, highlighting their advantages, challenges, and potential in biomedicine.^12,13^ However, the continuous evolution of scientific research and the expanding scope of COF-based theranostics warrant updated and comprehensive insights. While existing reviews cover the applications of COFs broadly, a review specifically targeting theranostic applications provides a more detailed and targeted analysis of the role of COFs in combining therapy and diagnostics. It can delve deeper into design strategies, functionalization methods, and the integration of therapeutic and diagnostic functionalities within COFs. The review can address recent challenges encountered in COF-based theranostics, such as improving stability, enhancing biocompatibility, scaling up synthesis methods, and addressing regulatory hurdles. Highlighting the most current strategies to overcome these challenges provides valuable guidance for researchers in the field.

**Figure 1:**
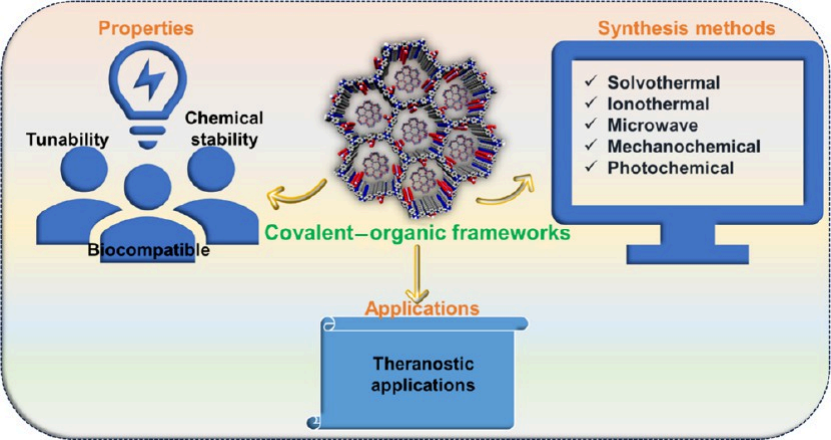
Schematic representation of properties, synthetic techniques, and applications of COFs.

In this review, we have systematically discussed the advances of COFs in the field of theranostics by providing essential features of COFs along with their synthetic methods, as outlined in Figure 1. In the next section, applications of COFs in the field of theranostics are discussed in detail with the help of available literature to date. Furthermore, the advantages of COFs over other materials for therapeutics and drug delivery are discussed. Finally, the review concludes with potential future COF applications in the theranostic field.

## 2 COFs, Their Synthetic Methods, and Properties

COFs are emerging crystalline porous organic polymers that have numerous benefits, such as large surface area, tunable pores, low density, and high thermal stability.^14^ At early stages, COFs have been explored in separation and gas storage.^15^ However, their modular nature opened numerous other applications, such as optoelectronics,^16^ biomedical sciences,^17^ and catalysis.^18^ COFs are connected through reversible covalent bonds instead of irreversible covalent bonds by which conventional polymeric materials are formed, which help to improve the crystallinity and biodegradability of COFs. For specific biomedical purposes, ample scope is offered to tailor COFs due to their scope of surface functionalization along with large ordered pores.^19,20^ In this section, we elucidate synthetic methods and their essential features of COFs, which benefit theranostic applications.

### 2.1 Synthesis Techniques for COF-Based Nanomaterials

In 2005, COFs were first synthesized and attracted researchers’ keen interest in this field. COFs are prepared solely by utilizing lightweight elements linked together through covalent bonds, resulting in robustness and a very low density of COFs.^21^ For the synthesis of COF materials, the main focus is on structural regularity, functionality, and porosity. To control and maintain the porosity, a proper design strategy is required. Several reports have been studied where the precursor’s solution utilized the porous template to produce pores in the material.^22^

**Figure 2:**
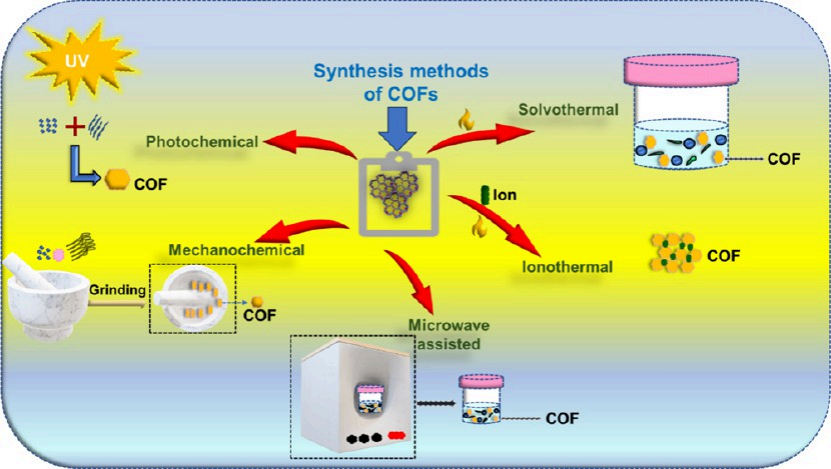
Various strategies for the synthesis of COFs.

Various strategies for synthesizing COFs, as showcased in Figure 2, are illustrated individually in this section.

#### 2.1.1 Solvothermal Synthesis

The solvothermal technique is the most commonly utilized for preparing COFs. This method provides a high temperature of about 70–120 °C to the reaction mixture involving precursor monomers, solvents, and catalysts/modulators in a closed vessel. Precipitates are obtained after the completion of the reaction, which were collected and washed with appropriate solvents. Powdered COFs are obtained after drying.^23^ The reaction’s temperature significantly influences the properties of the COFs, specifically the material’s crystallinity. For example, most of the B–O-linked COFs such as COF-6, COF-8, COF-10, COF-102, COF-103, COF-105, and COF-108 can be formed at 85 °C.^24^ Schiffs base reaction-based COFs are generally formed at 120 °C,^25^ while polyimide-based COFs are formed at higher temperatures like PI-COF-4 and PI-COF-5 at 160 °C,^26^ PI-COF-1 and PI-COF-2 at 200 °C, and PI-COF-3 at 250 °C.^27^ The solvent also strongly influences the COFs’ growth, and the crystallinity of COFs as solvent affects the solubility of the reactants. For instance, Gao et al.^28^ synthesized tetraphenyl ethene core-based COFs named TEP-COF-I and TEP-COF-II. The [4 + 4] condensation pathway was followed by the reaction when 15:15:2, v/v/v, of *o*-dichlorobenzene/*n*-butanol/acetic acid was utilized as a solvent, resulting in the fully bonded network (TPE-COF-I). In contrast, an unusual [4 + 2] pathway was followed when 1,4-dioxane/acetic acid (15:1, v/v) was used as a solvent which forms TPE-COF-II.

The solvothermal approach for forming COFs is a thermally controlled reaction, so the reactive sites of the monomers were protected with a protecting group in advance during the fast reactions between monomers to avoid the formation of amorphous polymers. This strategy can quickly obtain highly crystalline COF materials and reduce reaction rates.^29^ To optimize solvothermal kinetics and thermodynamics, multicomponent reactions are proven to be an effective strategy which provides crystallinity, precision, and a higher level of complexity to the covalent assembly.^30^ Wang et al.^31^ synthesized imidazole-linked COFs via the Debus–Radziszewski multicomponent reaction from *tert*-butylpyrene tetraone, aldehydes, and ammonium acetate under solvothermal conditions. The imidazole linkages in the synthesized material provide ultrahigh chemical stability, high crystallinity, and high thermal stability up to 400 °C to COFs. Generally, powered forms of COFs can be obtained by solvothermal methods, which restrict their applications in some circumstances.^22^ However, the solvothermal method has produced COFs as thin films in the past few years.^32^

#### 2.1.2 Ionothermal Synthesis

When the synthesis is carried out in ionic liquids (ILs), the process is known as an ionothermal synthesis. ILs are molten salts with melting points less than 100 °C and have been considered environmentally friendly solvents, making them promising materials for industrial applications.^33^ Thomas and his team synthesized porous crystalline COFs via an ionothermal technique.^34^ A mixture of molten ZnCl_2_ and nitrile was heated at 400 °C, which affords the covalent triazine-based framework (CTF), where ZnCl_2_ acts as a solvent and catalyst in a reversible cyclotrimerization reaction. However, this composite CTF material has the disadvantage of crystallinity control compared to COF materials prepared with a solvothermal approach due to the applied harsh reaction conditions for cyclotrimerization reactions. 3-D COFs were prepared by Guan et al.^35^ by utilization of 1-butyl-3-methylimidazolium bis((trifluoromethyl)sulfonyl)imide which acts as both solvent and catalyst in the Schiff base reaction. The COF material was produced under ambient temperature and pressure conditions. The surface area of obtained 3D-IL-COF-1, 3D-IL-COF-2, and 3D-IL-COF-3 was 517 m^2^/g, 653 m^2^/g, and 870 m^2^/g, respectively.

#### 2.1.3 Microwave Synthesis

Microwave synthesis methods accelerate the reaction rate and require less time for the preparation of porous and crystalline COFs. Generally, a mixture of suitable monomers and solvent is sealed under vacuum in a microwave tube and heated at a designated temperature with stirring for the appropriate time. Boron-based COF-5 was synthesized by a microwave technique where the reaction takes place at 65 °C, and the COF formed within 20 min. The authors compared the reaction time with the reported solvothermal method and found that the COF formed nearly 200 times faster than with the solvothermal method, where the COF was formed in 72 h.^36^ The surface area of the obtained COF was 2019 m^2^/g. COF better porosity can be obtained. The microwave method can remove oligomers from COF very efficiently.^37^

Furthermore, enamine-linked T_p_P_a_-COF was prepared by Wei et al.^38^ using the microwave synthesis method. Briefly, 1,3,5-triformylphloroglucinol (Tp) and *p*-phenylenediamine (in a 2:3 molar ratio) were mixed in a solution of mesitylene/1,4-dioxane/acetic acid and sealed in a microwave tube under vacuum. The reaction took place at 100 °C with stirring for 60 min, and the resulting solution was filtered and washed with acetone and mesitylene. Further, this method is suitable for the formation of 2D as well as 3D COFs^36^ and has been explored for the formation of ketoenamine,^39^ melamine,^40^ and triazine^41^ based COFs.

#### 2.1.4 Mechanochemical Synthesis

The conversion of mechanical and chemical energy in biochemistry utilized in physiological operations gives the origin to research in mechanical chemistry. Mechanochemistry provides mechanical energy via shearing, friction, and squeezing to induce chemical changes between solid materials.^6^ High-energy grinding equipment has been developed with the advancement of the machinery industry, which enabled mechanical chemistry’s application in different fields like organic and inorganic synthesis, compound modification, and metal alloying. For the preparation of COFs mainly, four types of mechanical synthesis equipment named ball milling, mortar, 3D printer, and extruder have been developed. This method is appealing due to its simplicity, as any harsh condition is not required to form the material.^23^

By manual grinding in a mortar and pestle, the β-ketoenamine-linked COF was first synthesized by Banerjee and co-workers in 2013. The visual change in the color was observed after 45 min, when the mixture turned dark red, indicating the complete formation of the COF.^42^ The chemical stability of the synthesized COF was almost the same compared with that of the COF synthesized via the solvothermal method. However, the crystallinity of the prepared COF was unsatisfactory. Further, to enhance the efficiency of the synthesis, grinding was performed. For instance, Wang and his team developed a COF called TpAzo via mechanochemical grinding of 4,4′-azodianiline (Azo) and 1,3,5-triformylphloroglucinol (Tp) with *p*-toluene sulfonic acid (molecular organizer) through a Schiff base aldehyde-amine condensation reaction. The synthesized COF had a surface area of 636 m^2^/g.

Liquid-assisted grinding (LAG) alleviates the material’s crystallinity within a short time; for instance, Β-ketoenamine and hydrogen-bonded imine-linked COFs were fabricated by LAG.^43^ Covalent–organic nanosheets can also be synthesized from the COF via mechanochemical synthesis. The as-synthesized COF was ground with a mortar and pestle for 30 min at room temperature. Then, 100 mL of methanol was added and centrifuged at 8000 rpm for nearly 5 min, forming a clear solution.^44^ Extrusion is another method for the mechanochemical synthesis of COFs. Karak and his team^45^ fabricated a COF by a screw extruder and terracotta process within 60 s. For the COF, initially Tp was added in *p*-toluene sulfonic acid and ground well. After that a small amount of water was added to the mixture and heated at 170 °C for 1 min; a dark reddish powder was obtained, dipped into hot water to separate the desired COF. The obtained COF exhibited a surface area of 3109 m^2^/g. With this approach, large-scale production of nearly some kilograms per hour of COFs is possible.

**Figure 3:**
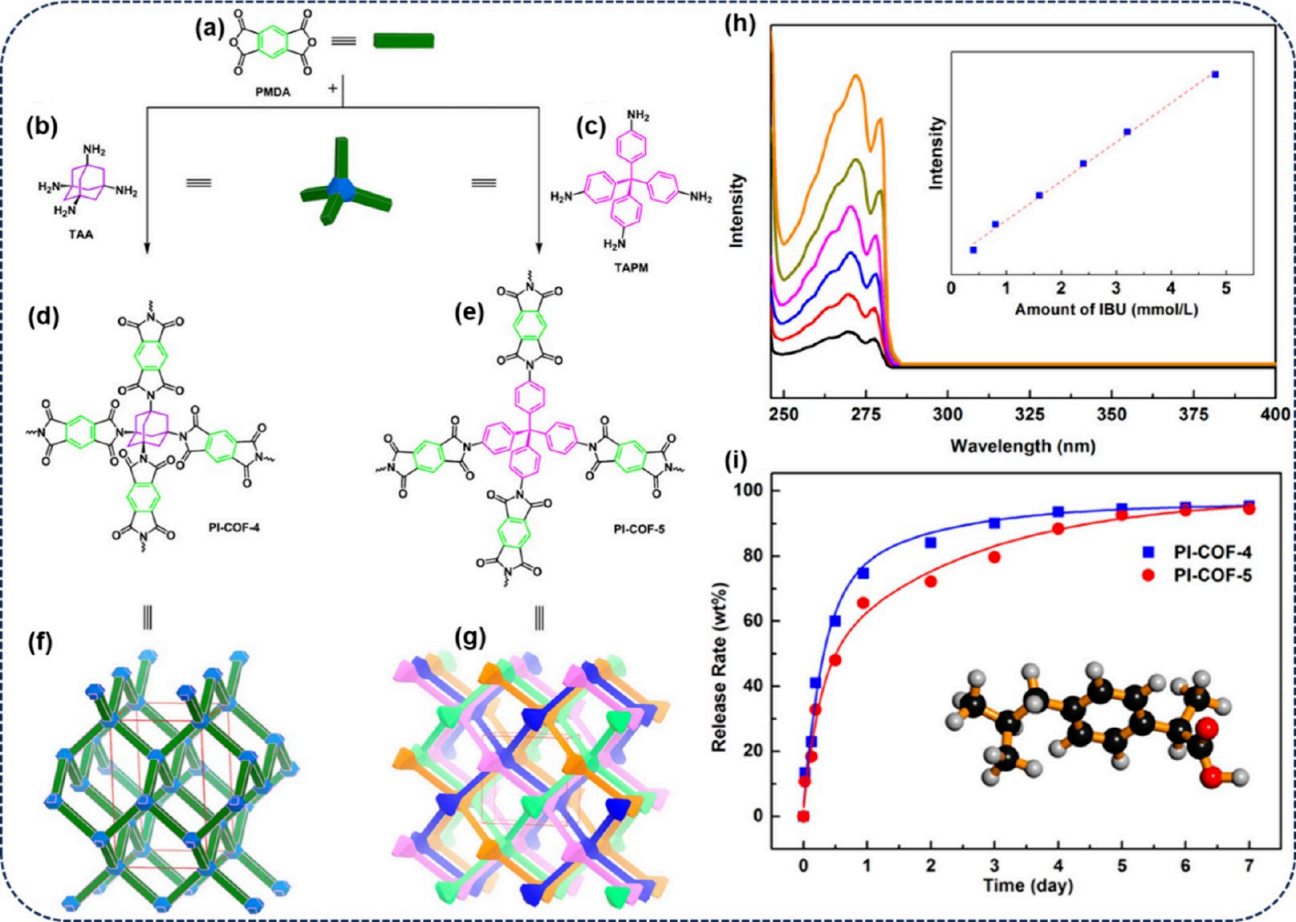
(a–g) Process for designing 3D spongy crystalline polyimide COFs (PI-COFs). (h) UV–vis spectra of ibuprofen (IBU) in simulated body fluid (SBF) were recorded at distinct concentrations. Inset: IBU calibration arc. (i) Discharge shapes of IBU-doped 3D PI-COFs. Inset: the design of IBU. C, black; H, gray; and O, red. Reprinted with permission from ref (26). Copyright 2015. American Chemical Society.

Fang et al.^26^ studied new 3D polyimide COFs with increased thermal strength and exterior area, assigned PI-COF-4 and PI-COF-5, and developed and prepared them through blending tetrahedral and linear structure units through the imidization reaction. Their design process for 3D absorbent PI-COFs is based upon a diamond grid with tetrahedral vertices. Via imidization, the linear coupling unit, pyromellitic dianhydride (PMDA) (Figure 3a), responds with the tetrahedral structure blocks 1,3,5,7-tetraaminoadamantane (TAA) (Figure 3b) and tetra(4-aminophenyl) methane (TAPM) (Figure 3c) to create the vast 3D framework arrangements PI-COF-4 (Figure 3d) and PI-COF-5 (Figure 3e), correspondingly. In the dawn of the various measures of TAA and TAPM and their productive bisimide connections, non- and bear-saturated diamond nets were predicted and marked here for PI-COF-4 and -5, correspondingly (Figure 3f,g). UV–vis spectrophotometry indicated that the drug discharge shapes utilize a calibration arc for ibuprofen (IBU) (Figure 3h,i). Corresponding with PI-COF-4, PI-COF-5 with a smaller pore size and saturated system indicates a lower discharge rate (e.g., 60% for PI-COF-4 vs 49% for PI-COF-5 after 12 h), which suggests that the drug delivery in COFs is instantly connected to the pore size and geometry. For both PI-COFs, most of the IBU was removed after about 6 days, and complete delivery would come at ca. 95% of the primary IBU loading.

A more recent method for mechanochemical synthesis is 3D printing. Raw materials and Pluronic F127 were mixed with the help of a 3D printing template, which resulted in the formation of hydrogels. After that, commercial 3D printers were utilized to produce COFs.^46^ Very delicate COFs were obtained by 3D printing due to their high operational accuracy and controllability. For the synthesis of COFs, the mechanochemical approach is still in a vigorously growing stage.

#### 2.1.5 Photochemical Synthesis

Photochemical synthesis methods have been utilized as one of the efficient and important synthetic routes to produce COFs quickly.^47^ High-energy intermediate states, which are difficult to make and utilize by thermal processes, can be easily accessible by photochemical processes. In addition, the photochemical route provides control over the reaction by changing the size of the beam or by applying optical masks, where using a laser instead of an ordinary light source helps change the beam’s size. Because of its less complicated route, the photochemical method can reduce irreversible damage caused by multistep procedures, such as solvent treatment and lithography.

Kim et al.^48^ developed uniform sea-urchin-shaped COF-5 under UV irradiations. The growth rate of the photochemical method was found to be nearly 48 times higher compared to that of the solvothermal counterpart. The as-synthesized COF exhibited a surface area of 2016.9 m^2^/g, and a 75% yield was obtained. A pyrazine-fused COF (hcc-COF) was synthesized at room temperature by Choi and his team by implementing a synthetic photochemical technique,^49^ and hcc-COF was prepared within 3 h by a condensation reaction. The authors confirmed the importance of light irradiation via the synthetic process in the absence of light, where only amorphous polymers were produced—the generated hcc-COF exhibited regular structure and 2.22 × 10^–3^ S/m of electrical conductivity. Liang et al.^50^ fabricated a cladding sea cucumber-like COF via the photochemical method. COFs with unique morphologies can be obtained by the utilization of photochemical routes in comparison to conventional methods.

**Figure 4:**
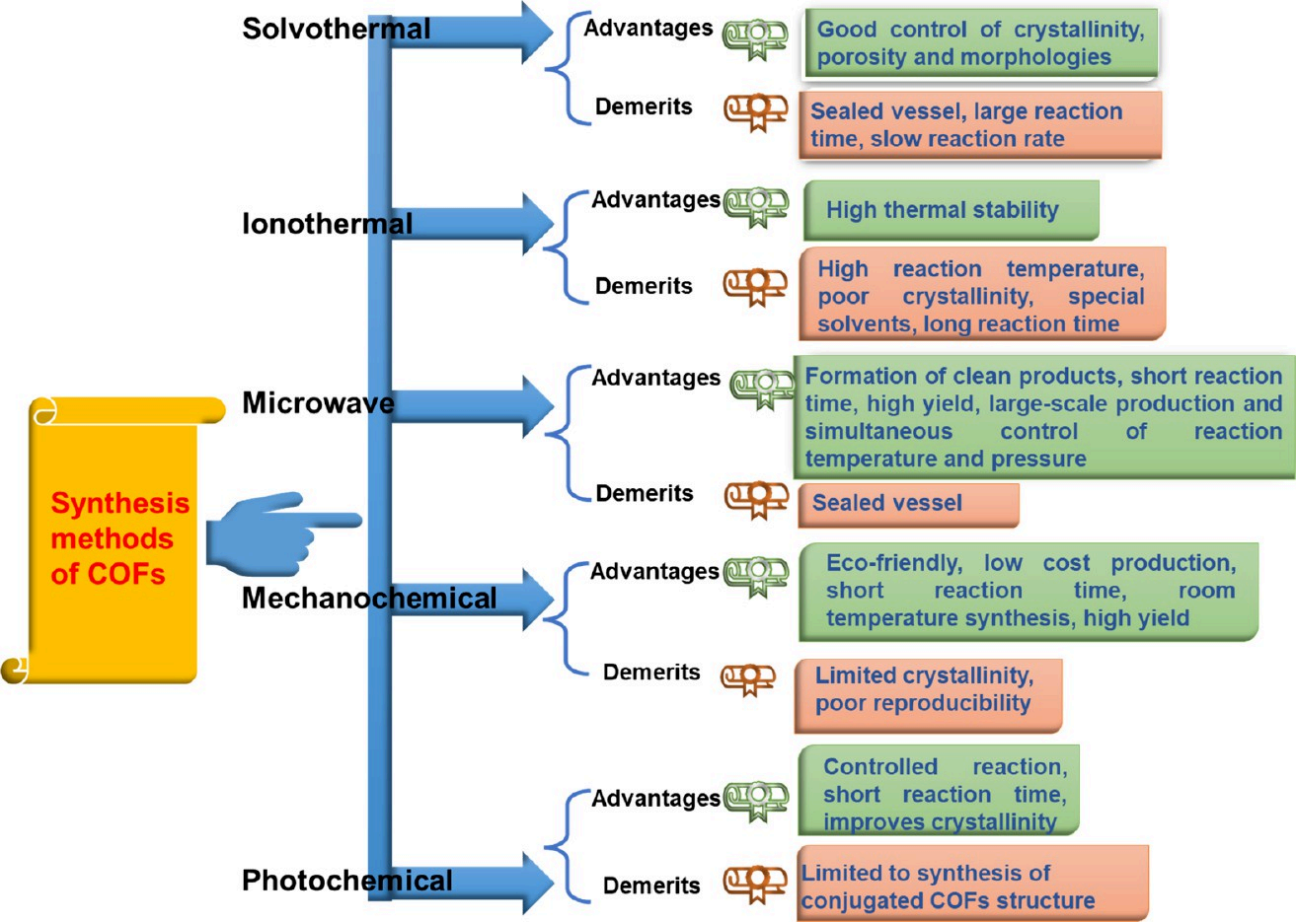
Advantages and disadvantages of different synthetic methods of COFs. Adapted from refs (19, 23, and 51). Copyright 2021. Elsevier. Copyright 2022. John Wiley and Sons.

The advantages and disadvantages of the above-mentioned synthetic methods of COFs are showcased in Figure 4.^19,23,51^

The synthesis of COF-based nanomaterials offers numerous advantages, such as versatility, precise control, and ecofriendliness, but it also presents challenges related to achieving high crystallinity, scalability, stability, and characterization. Overcoming these challenges is essential to unlocking the full potential of COF nanomaterials for various applications in catalysis, sensing, drug delivery, and optoelectronics.

### 2.2 Properties of COFs

The porosity, arrangement, shape, composition, and size of the pores are properties of porous materials that impact the material’s performance in different applications. Manipulation of the structure and the composition of porous materials have attracted the focus of scientists as these characteristics specify the function and control access of the internal surface of the material.^52^ Development in modeling and prediction of the skeleton of substructures and their associated units result in considerable enhancement of porous materials consisting of metal and COFs. Light elements are covalently bonded to carbon atoms. Independent adjustment and tunability of the pore geometry and chemical functioning with an accuracy of individual parameters are the modular characteristics of these materials.

Previously, this simultaneous control of the synthetic process and the combination was not possible with any material. Research is still in progress for manipulating the structures and fundamental characteristics of these materials to further enhance their performance. COFs are fabricated by light elements, so they are expected to provide high gravimetric performance for guest molecules.^53^

COFs exhibit high surface area, crystallinity, porosity with open pores, and low density, making them promising drug delivery materials. COFs have higher surface area in comparison to most porous materials like carbons and mesoporous silica.^54^ The surface areas for 2D and 3D COFs exceeded 3000 and 5000 m^2^/g, respectively. The capability of the material to resist decomposition and maintain chemical/physical structures after heating is known as thermal stability. This property is highly dependent on bond strength, and COFs have robust bond strength, which results in the high thermal stability of COFs.^55^ However, the mechanical properties of the COFs remain undiscovered. Mechanical loading can considerably affect the physiochemical properties of the materials. So, understanding the mechanical properties of COFs plays a significant role in their future successful applications. Table 1 summarizes the different properties of COFs.

**Table 1:** Different Properties of COFs

COFs	Linkage	Surface area (m^2^/g)	Pore size (nm)	Pore volume (cm^3^/g)	Thermal stability	Ref
COF-5	Boronate ester	1670	2.7	1.07	-	(56)
COF-202	Borosilicate	2690	1.1	1.09	450 °C	(57)
TpPa-1	B-ketoenamine	535	1.25	–	350 °C	(58)
TpPa-2		339	1.35	–		
COF-JLU6	Triazine	1450	3.1	0.96	-	(59)
COF-300	Imine	1360	0.72	0.72	490 °C	(15)
1,3,5-Tris(4-bromophenyl)benzene-COF	C–C	-	-	-	400 °C	(60)
COF-42	Hydrazone	620	2.3	0.31	280 °C	(61)
COF-43			3.8	0.36		
Py-Azine COF	Azine	2723	2.3	-	250 °C	(62)
Polyimide-COF	Imide	2403	1.0–1.3	-	450 °C	(26)
Cage-COF-triammonia-terephthalaldehyde	C–C	672	1.0	0.52	-	(63)

COFs possess unique properties that make them attractive for diverse applications. Structural stability, proper optimization, scalability, and characterization issues must be resolved to fully realize their potential in various fields, from energy storage to biomedicine. Ongoing research aims to overcome these challenges and further expand the applications of COFs in cutting-edge technologies.

### 2.3 Comparison of COFs with Other Commonly Used Materials in Theranostic Applications

**Figure 5:**
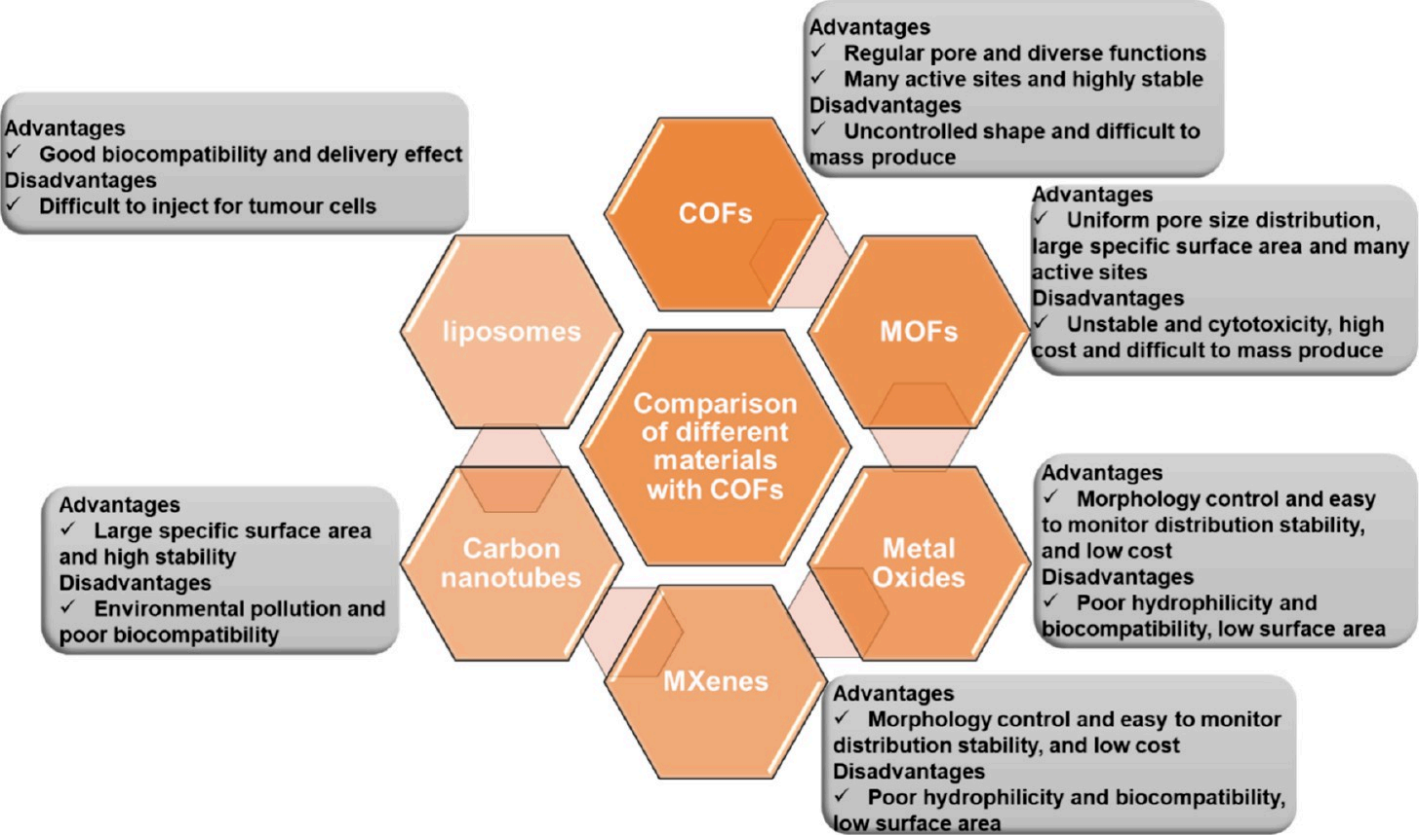
Pictorial represents the comparison of COFs with other commonly utilized materials in terms of pros and cons. Data adapted from refs (23 and 37). Copyright 2021. Elsevier. Copyright 2020. American Chemical Society.

The unique features of COFs that make them attractive compared to other porous materials are structural regularity, porosity, and atomic connectivity in their framework. COFs have high thermal stability up to 250–450 °C.^37^ Robust covalent linkage results in excellent resistance compared to most MOFs.^55^Figure 5 corresponds COFs with other commonly utilized materials regarding pros and cons.^23,37^

COFs possess distinct advantages, such as tunable structures, high stability, and potential biocompatibility, making them promising materials for theranostic applications. However, the choice between COFs and other materials depends on specific application requirements, considering factors such as biocompatibility, stability, scalability, and specific functionalities in theranostics. Integrating the strengths of these materials could lead to more advanced and effective theranostic platforms.

## 3 Applications of COFs in Theranostics

Due to unique features and significant potential in treatment and diagnosis, COFs have been used in photothermal and photodynamic therapies (PTT and PDT), bioimaging, drug delivery, and biosensing. Although COFs are in their early stages in TERM, further exploration is still required. Here, we highlight the potential applications of COFs in theranostics and expect that it will motivate and inspire more researchers to work with COFs toward future clinical translation.

### 3.1 COFs as Drug Delivery Systems

Generally, the requirement of a targeted system includes good loading efficiency with sustained and controlled release of payloads; carriers should be nontoxic in nature; and modification of the surface of nanocarriers must be possible.^64^

The dominant method for clinical cancer treatment is chemotherapy, and most chemotherapeutic drugs have specific limitations such as low chemical stability and solubility. Platinum-based anticancer drugs have poor photostability; cisplatin is one example that must be protected strictly during injection from light. However, complete negligence of photoredox and photohydration reactions is not possible. These photoreactive products pose higher side effects than cisplatin.^65^ Paclitaxel is an example of a drug with low water solubility, which results in restricted bioavailability and leads to more need for a drug supply to keep the concentration within the therapeutic range, hence surging the chances of off-target toxicity. Doxorubicin (DOX) has been criticized for cardiotoxicity, while digestive and renal toxicity can be caused by platinum-based anticancer drugs. These conventional drugs have some proportion of drug-resistant cells that interfere with achieving the desired results.^66^

Nanoparticle-based drugs have overcome the challenges faced by traditional anticancer drugs at the organ and tissue level. Three main characteristics, passive targeting, phagocytosis escape, and active targeting, are the main biodistributive features of nanoparticles. Phagocytosis escape helps to surge the active drug’s circulation time, which enhances the drug’s half-life.^67^ Passive targeting enhances the accumulation at the tumor site due to nanoparticles’ retention effect and high permeability. Furthermore, nanomaterials can be functionalized with antibodies,^68^ targeting groups,^69^ and aptamers^70^ to promote the interactions between tumor cells and membrane receptors, leading to endocytosis, which is known as active targeting. By endocytosis, cells take up the nanoparticles and therefore overcome the restrictions of small-molecule drugs toward selective cell membrane permeability. Nanoparticles are entrapped in the acidic organelles after being taken up by cells and then degraded slowly.

However, COFs are beneficial for mitigating the side effects, reducing drug doses, and enhancing the advantages of therapeutics. COFs, specifically imide-linked COFs, cause an increase in the pH of endo-/lysosomes via alkaline N-atoms of linkage; this increased pH induces the production of water and Cl– ions in these organelles. When the self-adjusting capacity of endo-/lysosomes is exceeded, they eventually rupture and release COFs into the cytoplasm.^71^ COFs have emerged as effective nanocarriers for antitumor drug delivery, including drug loading, controlled drug release, and targeted drug delivery.^72^

**Figure 6:**
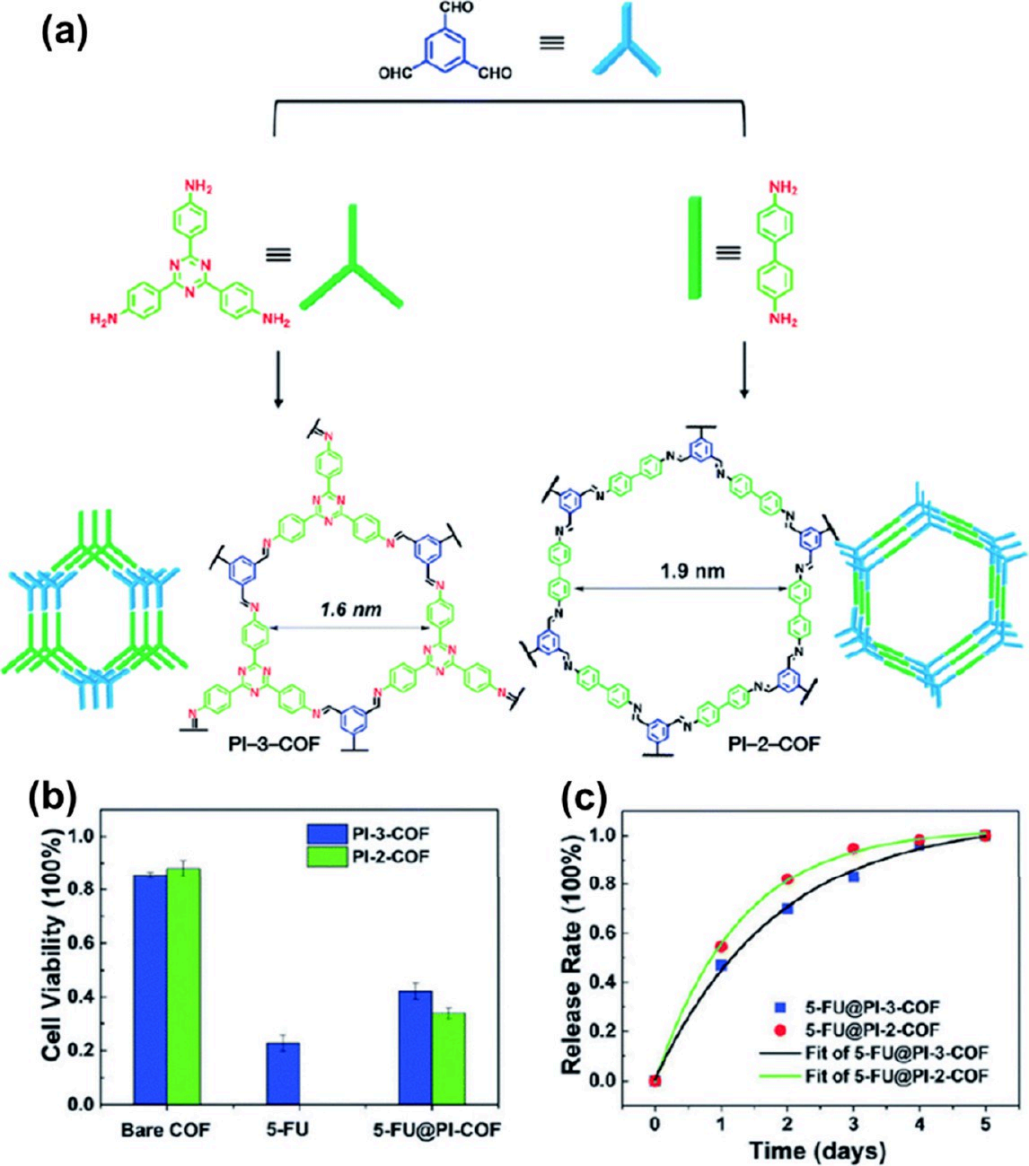
Synthetic procedure along with the topologies of PI-2-COF and PI-3-COF. Reprinted with permission from ref (6). Copyright 2020. Royal Society of Chemistry.

Zhao and team began COF-based drug delivery in 2016. They studied the two imine-based COFs named PI-2-COF and PI-3-COF as drug carriers to observe their drug release and loading capacity. Also, they studied in vitro cytotoxicity, as showcased in Figure 6a.^73^ The authors showed that the synthesized COFs exhibited good biocompatibility. PI-2-COF and PI-3-COF were stirred with fluorouracil (5-FU) in *n*-hexane for drug loading. The cell viability of synthesized material reduced to nearly 40% after 24 h of incubation with MCF-7 breast cancer cells, and the effect of surface modification of 5-FU is showcased in Figure 6b. The drug release curve, as showcased in Figure 6c, described that 5-FU@PI-2-COF and 5-FU@PI-3-COF provided the capacity of continuous drug release for several days. However, the cytotoxicity of these COFs was weaker than free 5-FU within 4 h.^6^

Another imine-based COF (TTI-COF) was prepared by Vyas et al.^74^ via the reaction of triazine triphenylamine (TT-am) with triazine triphenyl aldehyde (TT-ald). Imine has a nitrogen center with free electron pairs that harbor the guest molecules through noncovalent hydrogen linkage. A dietary flavonoid named quercetin is known for its anticancer and antitumor features and can provide H-bonding between hydrogen atoms of quercetin’s hydroxyl group and nitrogen atoms of imine within COFs.

This linkage type is advantageous for targeted drug loading and release by anchoring guest drug molecules. So, TTI-COF was investigated as a drug carrier in cancer cells for the delivery of quercetin. On human breast carcinoma MDA-MB-231 cells, *in vitro* experiments were performed, and authors found that quercetin-loaded TTI-COF effectively kills the cancer cells within 4 days without affecting normal cells. In another study, PI-COF-4 and PI-COF-5 were fabricated by the imidization reaction between pyromellitic dianhydride and 1,3,5,7-tetraaminoadamantane/tetra(4-aminophenyl)-methane. The synthesized PI-COF-4 has a pore size of 13 Å, and PI-COF-5 has a pore size of 10 Å. Ibuprofen (IBU) can be easily entrapped by PI-COFs, and the molecular size of IBU is 5 × 10 Å. The drug release efficiency after 12 h was 60% and 49% for PI-COF-4 and PI-COF-5, respectively.^26^

**Figure 7:**
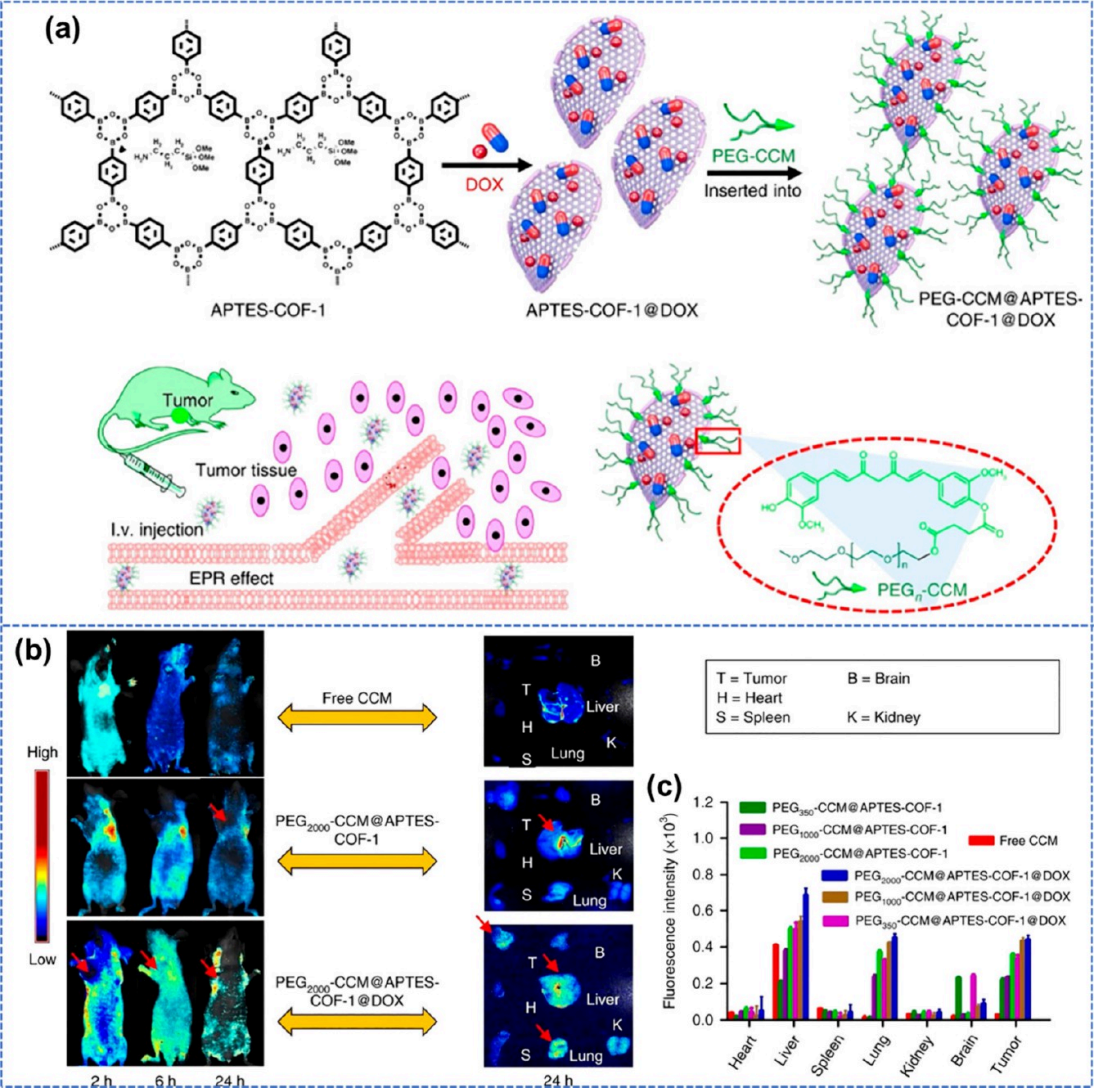
(a) Synthetic representation of 2D COFs including DOX delivery *in vivo*. (b and c) Fluorescence images and intensities of major organs and tumor of mice after injecting synthesized materials. Reprinted with permission from ref (76). Copyright 2023. American Chemical Society.

The COFs mentioned above were investigated as drug delivery carriers *in vitro* due to certain restrictions, such as low dispersibility, which result in a lower bioavailability of COFs with cells. This issue was overcome by Zhang et al.^75^ by reporting water-dispersible polymer COFs as drug delivery carriers for both *in vivo* and *in vitro* applications. Figure 7 represents the synthetic procedure where, first, 3-aminopropyltriethoxysilane-functionalized COF-1 (APTES-COF-1) was utilized for the loading of DOX to produce nanomaterial APTES-COF-1@DOX which further undergoes self-assembly pegylated (PEG) curcumin (CCM) for the generation of the final nanocomposite PEG-CCM@APTES-COF-1@DOX.

PEG-CCM@APTES-COF-1 showed robust fluorescence, which helps trace DOX’s release and cellular uptake on the COF. The drug loading capacity of the as-synthesized nanocomposite was 9.71 ± 0.13 wt % with an attractive encapsulation efficiency of 90.5 ± 4.1%, higher than APTES-COF-1. *In vitro* experiments revealed that DOX-loaded PEG-CCM@APTES-COF-1 inhibited the growth of HeLa (cervical carcinomas) cells even with a minimal concentration of DOX. This excellent *in vitro* performance inspired the authors to perform *in vivo* experiments with a xeno-grafted tumor model of HeLa cells on nude mice. The results showed that the as-prepared nanocomposite reached the tumor site efficiently with prolonged circulation in the bloodstream, with higher anticancer efficiency (Figure 7b and c).^76^

Liu et al.^77^ also prepared PEGylated COFs (F68@SS-COFs), which effectively load and deliver DOX to kill tumor cells. The as-synthesized material’s half inhibitory concentration value was nearly 3.62 μg/mL, higher than free DOX (1.78 μg/mL). Further, the authors observed that F68@SS-COFs exhibited a higher distribution of DOX, enhancing its cell-killing activity. Many reports have been conducted on the preparation of COFs and their utilization in drug delivery. Still, only a few studies investigated the detailed research for applications of COFs *in vivo* and *in vitro*. In addition, toxicity and specific targeting must be considered in depth for practical applications.

### 3.2 Photodynamic Therapy

Photodynamic therapy (PDT) is a noninvasive therapeutic technique for cancer treatment and exhibits beneficial attributes such as fewer side effects and feasible operation. Therefore, it can be considered a potential cancer treatment alternative.^78^ The key features on which PDT relies are photosensitizers (PSs), light of a particular wavelength, and oxygen.^79^ Numerous photosensitizers are available, such as cyanides, porphyrins, derivatives of xanthene, acridine, chlorophyll, and boron-dipyrromethene (BODIPY), which serve the role of therapeutic agents and result in the formation of reactive oxygen species (ROS) by interacting with oxygen and light. Specifically, cytotoxic singlet oxygen is formed, which destroys cancer cells. However, these conventional PSs have limitations of poor water solubility, lower cell permeability and aggregation tendency, and lower performance of PDT in terms of selectivity and efficiency.^13^ When PSs are modified with COFs, fluorescence quenching can be prevented, and quantum yields of ROS can be enhanced. Significant benefits of PDT are its selectivity to kill the targeted tumor cells without any detrimental effects on healthy organs; it can be utilized in low doses for reducing the adverse effects that are caused by the nonresisting toxicity of ROS; and the light needs for PDT can be directed from fiber optics and can be combined feasibly with other cancer treatment methods such as radiotherapy and cancer therapy.^80^

**Figure 8:**
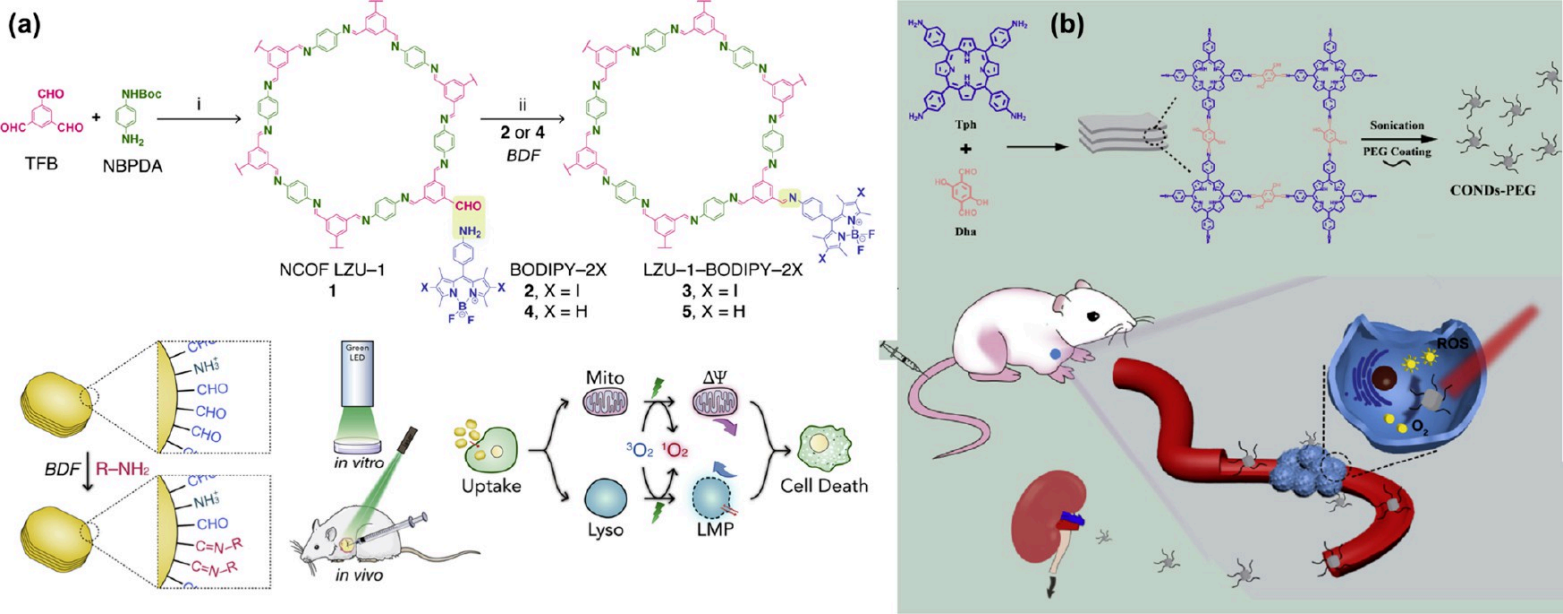
(a) BODIPY-modified COF for PDT with a synthetic procedure. Reprinted with the permission of ref (81). Copyright 2019. Elsevier. (b) Schematic representation of COF nanodots-PEG in PDT for cancer therapy. Reprinted with the permission of ref (82). Copyright 2019. Elsevier. Furthermore, a COF nanosheet-modified porphyrin photosensitizer (PcS@COF-1) was developed using π–π interactions.^83^ PcS@COF-1 showed excellent hydrostability and *in vivo* and *in vitro* restrained the prefiltration of tumor cells under light irradiation. In another study, COF-909 synthesized by Deng and co-workers can act as a photosensitizer with an outstanding production capacity of ROS and decent biocompatibility and photostability.^79^ The experiment was performed *in vivo* as well as *in vitro. In vitro* experiments demonstrated that a lower concentration of 50 mg/mL with infrared light irradiation of 630 nm COF-909 can kill almost 80% of cancer cells. The *in vivo* investigation of the PDT efficiency of COF-909 was done by injecting the synthesized COF into CT26-tumor-bearing mice, and no apparent growth of tumor cells was observed.

As COFs have been successfully utilized as drug delivery agents, it is logical that COFs can be used as PDT agents for cancer treatment. A team of researchers^81^ prepared BODIPY-based COF nanorods via bonding defect functionalization, as shown in Figure 8a. The prepared material had a smaller size of nearly 110 nm and exhibited high phototoxicity in *in vivo* and *in vitro* experiments. The authors observed that the synthesized nanocomposite serves as a potential PDT agent as it inhibits HeLa cells and MCF-7 and generates cytotoxic singlet oxygen in large amounts. A simple liquid exfoliation method was utilized to synthesize porphyrin-based COF nanodots modified with PEG by Zhang et al.,^82^ as described in Figure 8b. The size of the as-prepared COF nanorods was 0.22 nm and produced high ROS with decent biocompatibility and physiological stability after light irradiation. The experiment was performed on normal (RAW 264.7 and L929) and tumor cells (MDA-MB-231 and HeLa). The results revealed that the prepared nanocomposite had no considerable cell cytotoxicity even at high concentration (200 μg/mL). When this composite was applied to tumor-bearing mice, the size of the tumor of mice under light irradiation had no significant growth. These results demonstrated that COF nanorod-modified PEG had decent PDT efficiency and could be excreted by renal filtration without affecting other normal tissues.

### 3.3 Photothermal Therapy

Photothermal therapy (PTT) is another required phototherapy method where photothermal agents (PTAs) have been utilized for heat generation from light absorption, leading to elevated temperatures to kill tumors or cancer cells.^84^ The transfer of energy starts from light to electrons, which are further transferred to the lattice. Then the energy is transferred in the form of heat from the lattice to the environment, and this phenomenon is known as a photothermal effect. Numerous PTAs can efficiently absorb near-infrared (NIR) light, as they have a more extensive π-conjugated system and get excited. The thermal effect is induced by released energy via a nonradiative transition. Electromagnetic radiation excites PTAs with specific band light, and the heat released by these excited agents kills the tumor or cancer cells. There is no oxygen requirement (as needed for PDT) in PTT for the interaction with abnormal target cells.^85^

Several materials, such as semiconductor nanoparticles,^86^ metal plasmonic nanostructures,^87^ and conjugated polymers,^88^ have been studied for photothermal ablation of cancer and tumor cells. However, these materials suffer from poor biocompatibility and water solubility and are suitable for practical applications. Owing to good photostability, biocompatibility, and outstanding photothermal effect, COFs attract the attention of researchers. However, studies have yet to be reported regarding the photothermal characteristics of COFs and need more deep research to explore the PTT application of COF-based materials.

**Figure 9:**
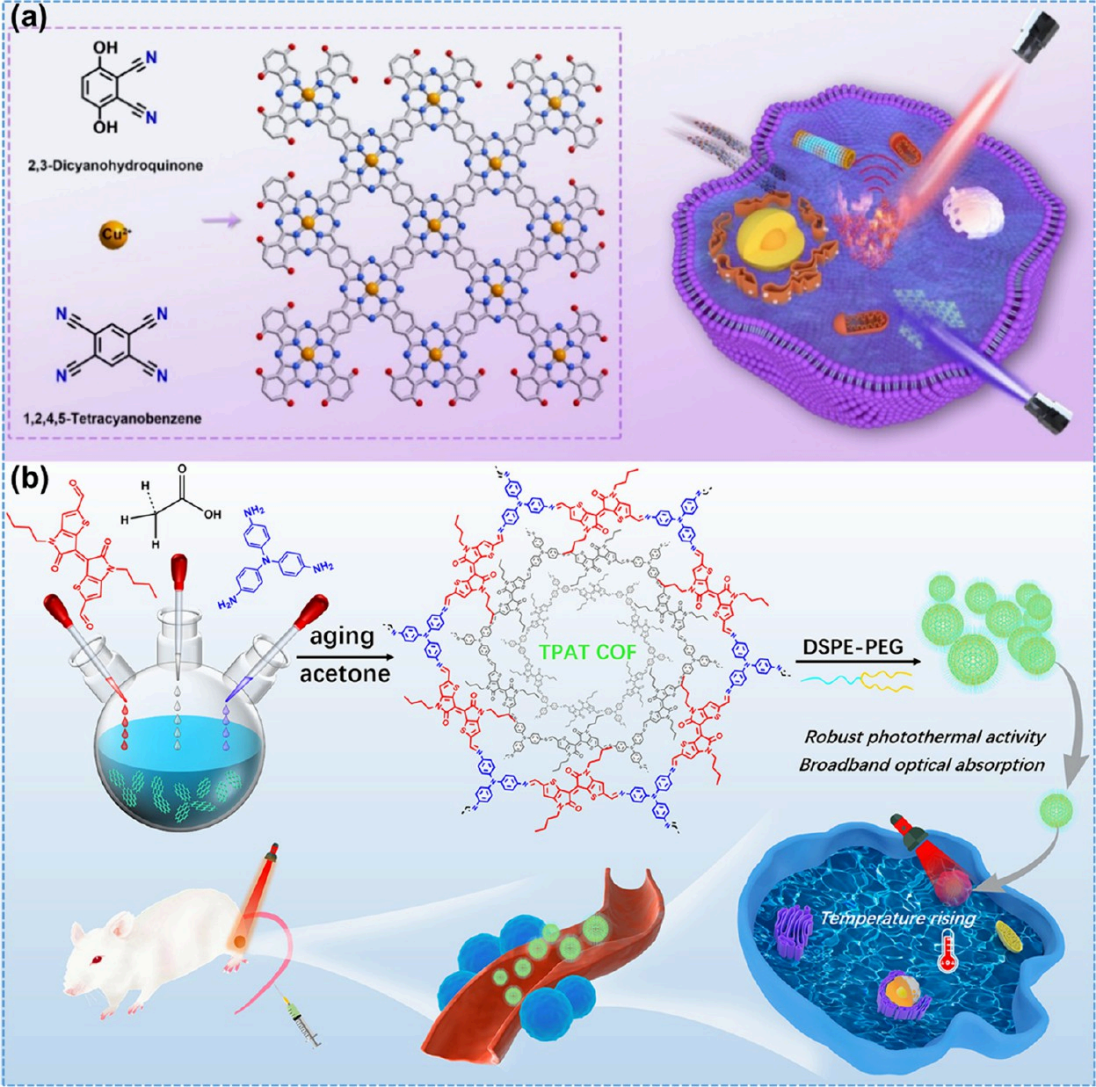
(a) Synthesis process of COF-Cu along with multicompatible therapeutics. Reprinted with permission from ref (89). Copyright 2021. Elsevier. (b) Preparation method of the TPAT COF and its PTT performance for tumor treatment. Reprinted with permission from ref (84). Copyright 2022. American Chemical Society.

The edge-confined technique was developed by Li et al.^89^ through hydroxylating advantages of COF (COF-Cu) demonstrated in Figure 9a. The as-prepared COF displayed superior water stability, enhancing biocompatibility and penetrability. Around 10.3% and 39.3% were obtained from the fluorescence quantum yield and PT conversion efficiency of COF-Cu, respectively. Furthermore, COF-Cu was stable after 5 cycles without reducing the conversion efficiency. Another team of researchers^84^ prepared the TPAT COF at room temperature and showed a considerable red-shift spectrum in the NIR region, as demonstrated in Figure 9b. The performance of PTT was examined by using 808 nm laser irradiation, resulting in a conversion efficiency of 48.2%. Moreover, the stability of the process was maintained for more than five cycles. Lower cell viability in HeLa cells was observed after laser irradiation for 5 min. At a concentration of 80 μg/mL, more than 80% of the cancer cells were killed. Radical cation-containing COF (Py-BPy+.-COF) was studied by Guo and co-workers via *in situ* reactions.^90^ The excellent PT conversion efficiency of 63.8% was observed with a light irradiation of 808 nm and 55.2% for a light irradiation of 1064 nm. After applying a synthesized nanocomposite, almost all the A549 (lung carcinoma) cells were destroyed under light irradiation for 5 min; *in vivo*, results were also in agreement with *in vitro* results.

### 3.4 Combined Therapies

**Figure 10:**
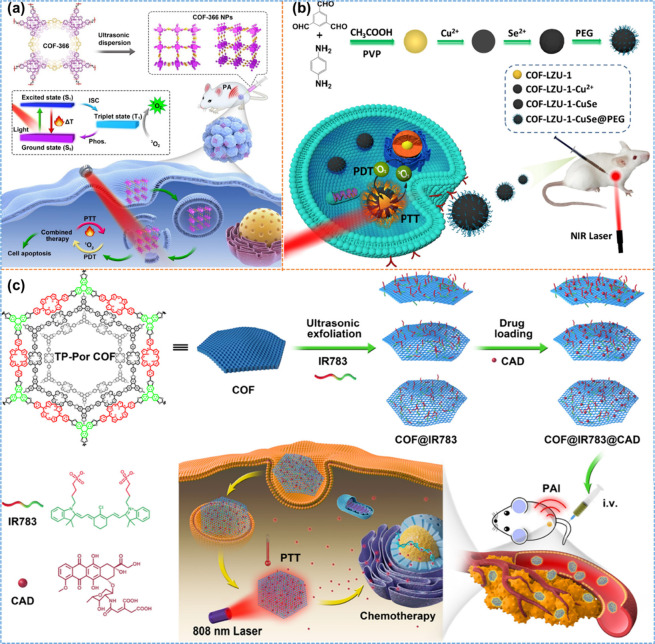
(a) Schematic illustration of COF-366 and photothermal therapy under light irradiation of a single wavelength. Reprinted with permission from ref (92). Copyright 2019. Elsevier. (b) Construction and PDT and PTT treatment of COF-CuSe. Reprinted with permission from ref (93). Copyright 2019. American Chemical Society. (c) Systematic representation of the synthetic procedure of COF@IR783 along with its combinative anticancer therapy *in vivo*. Reprinted with permission from ref (94). Copyright 2019. American Chemical Society.

PDT and PTT have been largely utilized in treating cancer and tumors; however, these monotherapies have some inherent shortcomings, such as complete degradation or challenging removal of tumor tissue. For enhanced therapeutic effects, these shortcomings of monotherapy should be avoided, for which a combination of therapies such as PDT and PTT, PDT and chemotherapy, etc., is an effective strategy. Combination therapy provides long-term remission, improves the chances of a cure, and mitigates detrimental effects on vital tissues and organs compared to monotherapy.^91^ Wang et al.^92^ utilized ultrasonic exfoliation of bulk COF-366 to prepare COF-366, as shown in Figure 10a. PS quenching was reduced by a regular arrangement of porphyrin monomers in the framework. When a laser of 635 nm was irradiated, COF-366 generated singlet oxygen effectively. The photothermal conversion efficiency of 15.1% was yielded with a 200 μg/mL concentration of COF-366 because of the broadened adsorption band of synthesized material due to its conjugated structure. Flow cytometry analysis revealed that the apoptotic rate of 4T1 cells was 70.4% under light irradiation. Furthermore, *in vivo* photoacoustic imaging displayed that within 1.5 h of intertumoral injection COF-366 nanoparticles were spread into the entire tumor and inhibited the complete growth of the tumor within 14 days.

Another study used combined PDT and PTT to treat cancer cells with a porphyrin-based COF prepared at room temperature.^93^ In brief, the facile solution-phase synthesis process of 1,3,5-triformylbenzene and 1,4-diaminobenzene results in the formation of COF-LZU-1. After that, an aqueous solution of Cu(NO_3_)_2_ was mixed with synthesized COF, and the COF-Cu^2+^ complex was produced due to the formation of coordinate bonds between Cu^2+^ and nitrogen-related functional groups of COF nanoparticles. Lastly, the reaction between COF-Cu^2+^ and freshly prepared Se^2–^ solution generates COF-CuSe, as showcased in Figure 10b.

The size of the prepared nanocomposite was 150 nm. The as-synthesized nanocomposite produced singlet oxygen under a light irradiation of 808 nm. COF-CuSe exhibited high photostability up to 200 °C, confirmed with on/off cycles, which were repeated 8 times, and the photothermal conversion efficiency was 26.34%. *In vivo* studies revealed that COF-CuSe efficiently and quickly converts light energy into heat as the temperature rises from 29.1 to 62.3 °C within 5 min after the injection of COF-CuSe.

Wang et al.^94^ prepared a cyanine-based COF (COF@IR783) through ultrasonic exfoliation. They tested it in the application of combination therapy, including chemotherapy and photothermal therapy, both *in vivo* and *in vitro*, as described in Figure 10c. When anticancer *cis*-aconityl-DOX (CAD) was loaded to COF@IR783, it efficiently impeded 4T1 tumor cells under light irradiation of 808 nm. The summary of COF-based materials for different therapeutic applications is illustrated in Table 2.

**Table 2:** Summary of the COF-Based Materials for Different Therapeutic Applications^a^

COFs	Linkage	Shape and size (nm)	Surface area (m^2^/g)	Therapeutics	Performance	Ref
PI-COF-4	Imine	-	2403	Drug delivery of IBU, caffeine, and captopril	Exhibited 24% and 95% drug loading and releasing capacity, respectively	(26)
PI-COF-5			Exhibited 20% and 95% drug loading and releasing capacity, respectively		1876	
PI-3-COF	Imine	Needle shaped and 1.1	1000	Drug delivery of IBU, 5 FU, and captopril	Both COFs showed maximum loading capacity of 30% and a drug release capacity of 85% toward FU	(73)
PI-2-COF		Spherical and 1.4	1700			
Fluorescent COF	Imine	Spherical and 200	-	Drug delivery of DOX	35% loading capacity toward DOX and intrinsic fluorescent features with pH responsiveness that monitor drug loading with the naked eye	(95)
F68@SS-COFs	Imine	Spherical and nearly 140	-	Drug delivery of DOX	Displayed loading capacity of 21%	(77)
PEG-CCM@APTES-COF-1	Boroxine	-	-	Drug delivery of DOX	Exhibited loading capacity of 9.71 ± 0.13% and encapsulation efficiency of 90.5 ± 4.1%	(75)
DT-COF	Imine	-	-	Drug delivery of carboplatin	Showed loading capacity of 31.32% with targeted drug delivery	(96)
TTI-COF	Imine	Flake-like structure	2197	Drug delivery of quercetin	Quercetin-doped TTI-COF displayed improved anticancer activity as compared to free quercetin	(74)
TAPB-DMTP-COF	Imine	200	1000	Drug delivery of DOX	Exhibits loading capacity of 32.1%	(97)
COF-909	Imine	-	2610	PDT	More than 80% of tumor cells were killed *in vivo*	(79)
Fe-hierarchical COF	Imine	Flower-like sphere and 2–4 μm	-	PTT	87.8% antitumor efficiency was shown for the *in vivo* treatment of cancer cells	(98)
PEG-based COF nanodots	Imine	3.46	-	PDT	Displayed outstanding PDT efficiency to the growth of tumor *in vitro* as well *in vivo*	(82)
Tph-DMTP-COP	Imine	Spherical	-	PDT/PTT	Showed photothermal efficiency of 32.88%	(99)
TpBD-COF	Imine	Spherical and 20–100	1346	PTT	Showed photothermal efficiency of 21.5%	(100)
BODIPY-COF	Imine	110	822	PDT	BODIPY acts as a photosensitizer under green light irradiation	(81)
COF-366	Imine	100	379.70	PDT/PTT	COF-366 acts as PDT and PTT agents	(92)

a TAPB: 1,3,5-tris(4-aminophenyl) benzene. DMTP: 2,5-dimethoxyterephthaldehyde. BD: benzidine.

### 3.5 COFs in Diagnosis

Early diagnosis plays an essential role in improving treatment methods for any disease. The survival rate of patients can be enhanced by the development of early diagnosis technologies. Owing to several unique features such as eclipsed π–π stacking structure, long-range crystal domain, and good biocompatibility, COFs have significant potential to be utilized as excellent carriers for tumor detection substances.^101,102^ Porphyrin-based COF (p-COF), a synthesized aptasensor created by Yan et al.,^103^ selectively and sensitively binds to the epidermal growth factor receptor (EGFR) to detect human breast cancer MCF-7 cells. For EGFR, the as-prepared receptor had a detection limit of 7.54 fg/mL over a wide linear range of 0.05–100 pg/mL, and for MCF-7 cells, 61 cells/mL was the detection limit with a detection range of 500 × 10^5^. This excellent performance of p-COF was mainly attributed to factors like 2D nanosheet structure, which provides more binding sites for aptamer strands, larger pore channels of p-COF, which surge the adsorption capacity of material, and high π-conjugated design that increases the interaction between analyte and COFs.

Colorectal cancer (CRC) can be detected using COF-based materials. Different methods like pathological biopsy and colonoscopy have been widely utilized for diagnosing CRC, but these methods have some definite drawbacks, such as lower relative survival of CRC patients, which is five years.^104^ Exosomes are biomarkers which are useful in the detection of CRC. The monitoring and diagnosis of exosomes make diagnosis of cancer easier.^102^ Wang et al.^105^ fabricated COF-based nanoprobes where COFs were functionalized with *para*-sulfocalix[4]arene hydrate (pSC_4_) modified gold nanoparticles (AuNPs) and horseradish peroxide (HRP). In this probe, AuNPs accelerate the charge transfer process. PSC_4_ works as an amicable linker that binds different amino acid residues on the surface of exosomes.

The obtained sensor detects CRC with a detection limit of 160 particles/μL with a wide linear range of 5 × 10^2^ to 10^7^ particles/μL. Nevertheless, there is a need to explore diagnosis of different diseases using COFs.

## 4 Advantages and Challenges of Using COFs in Theranostic Applications

### 4.1 Advantages of Using COFs in Theranostic Applications

COFs have numerous advantages that make them potential candidates in the applications of theranostics, which are described in this section.

#### 4.1.1 Biocompatibility

The material’s ability to generate or initiate appropriate biological response and its complete clearance without toxicity in a specific application is biocompatible.^106^ Numerous parameters, such as porosity, size, pore geometry, surface property, and morphology, play a crucial role in the material’s biocompatibility. So, in biological applications for assessing the material’s biocompatibility, the physical characteristics of the material should be evaluated carefully to estimate their interactions with cell organelles and blood. Biocompatibility depends on the biodegradation mechanism, metabolization, and nontoxicity of the material. The building blocks highly affect the nontoxicity of COFs, so fabricating the COFs with the utilization of building blocks with minimal toxicity can help to achieve long-term COFs.^107^ Further, the studies revealed that the exclusion rate depends on the mechanism of clearance or degradation of NPs and that the pH-based hydrolysis of COFs is comparatively faster than catalytic degradation with carbon nanotubes.^10^ However, information regarding the later stages of biocompatibility of COFs is still in the emerging stage.

Dynamic covalent bonds are utilized in COFs as linkages, which helps keep their structure in normal conditions, and are broken via simulation like acid.^108^ This property provides enough stability to COFs; before reaching the target tissue, their structure remains preserved and biodegrades as their task is finished. In addition, cargo can be released swiftly under simulation by COFs, especially those with imine linkages. It is also found that COFs possess pH responsiveness because of the protonation and deprotonation of triazine units.^109^ Consequently, COFs can be ideal therapy carriers due to their biodegradation and stimuli responsiveness.

#### 4.1.2 High Surface Area and Tunable Pore Geometry

Drug delivery or drug loading efficiency is highly dependent on porosity and pore geometry, as the size of the drug molecule should be comparable to the pore geometry for efficient drug loading. Suitable pore geometry and larger surface area provide high drug loading efficiency.^10^ The surface area and pore volume of the COFs are high because of their unique framework structure. These features of COFs are much better when compared to carbons and mesoporous silica.^54,110^ Furthermore, pore geometry can be adjusted by changing the building blocks; i.e., COFs have tunable pore geometry, which helps to carry and control different pharmaceutical agents and their release.

#### 4.1.3 Excellent Photoelectric Properties and Modifiability

Most COFs comprise a laminated structure and π-conjugated system, which enhances their photoelectric properties to a greater extent. For example, electron acceptors or donors such as porphyrins, phthalocyanines, and naphthylamides can feature the proton conductivity of COFs, and polycyclic aromatic units such as pyrenes and tetraphenyl ethenes endow COFs with fluorescence. This characteristic is suitable for bioimaging and biosensing. COFs possess tailorable building blocks, which are helpful even in producing singlet oxygen for PDT.^111^

### 4.2 Challenges Using COFs in Theranostic Applications

Porosity, repeatability, crystallinity, and nanoscale size of particles play a crucial role in the pharmaceutical field because drug loading capacity and their possibility of entering cells are directly related to these properties. However, these requirements cannot be achieved simultaneously with existing synthetic methods of COFs, and even large-scale production is not easily possible with the synthetic methods of COFs. This limitation of COFs largely influences their performance in practical theranostic applications. Therefore, new plans for the synthesis of COFs must be developed.

**Figure 11:**
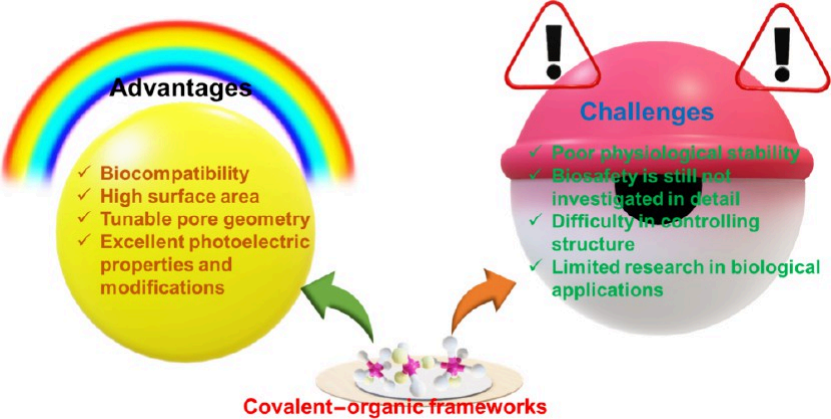
Graphical representation of different advantages and challenges of the COFs in theranostic applications. Information collected from ref (112). Copyright 2021. John Wiley and Sons.

Furthermore, the toxicity and biocompatibility of COFs still need to be fully explored. Although toxic metal elements are absent in COFs, several polycyclic aromatic derivatives consisting of multiple reactive groups are included in their degradation products. The effect of these groups on living beings is still unknown, and long-term toxicity *in vivo* requires much clarification, which is essential for their theranostic applications. Figure 11 represents the different advantages and challenges of COFs in theranostic applications.^112^

COFs are still in their infancy and have specific issues that need to be resolved compared with other materials. The first and most crucial issue is preparing COFs with high crystallinity, high porosity, and large specific surface area. In particular, the practical application of interested COFs heavily depends on their synthesis’s scalability. The design and synthesis of hydrophilic COFs are crucial because they are more stable in aqueous solutions and help with cellular uptake. Furthermore, most recent research focuses on how tumor cells absorb COFs; however, a thorough examination of the compound’s metabolic pathway still needs to be completed. Finally, a comprehensive assessment of their long-term toxicity is necessary because COFs have stable chemical properties that make them difficult to degrade.

## 5 Conclusion and Future Prospects

In conclusion, by outlining the key characteristics of COFs and their synthesis processes, we have methodically explored the advancements of COFs in the field of theranostics. Additionally, with the aid of the literature published to date, the uses of COFs in the field of theranostics—such as drug delivery systems and photothermal and photodynamic therapy—are covered in detail. This review thoroughly summarizes the various COFs associated with theranostic applications. The primary benefits of COFs that make them excellent candidates for biomedical applications are their larger surface area, easily accessible pores, tailored and ordered surface and structure, and biocompatibility. This new multifunctional therapeutic modality can significantly expand the potential of COF-based materials in biomedical applications. Moreover, it is noteworthy that most of the COF-based nanomotors reported to date depend on external stimuli, primarily light, for their propulsion.

**Figure 12:**
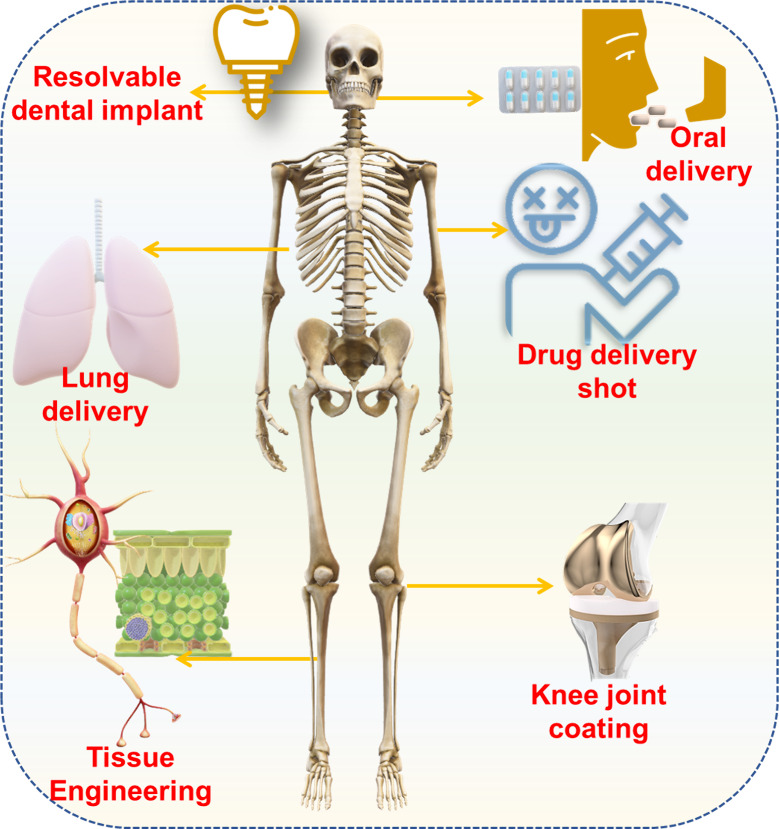
Some of the future applications of COFs.

However, despite many benefits, COFs have several limitations that restrict their practical applications. Limited physiological stability is one of the significant applications of COF materials. Polycyclic aromatic derivatives are primarily utilized in building blocks of COFs, which results in poor water dispersibility. So, developing COFs with high physiological stability is highly desirable even in harsh conditions. Figure 12 showcases some of the future applications of COFs.

Over the last couple of years, several types of research have been done, focusing on developing new building blocks and linkage motifs of COFs to enhance synthetic strategies, pore space, stability, and geometry to achieve scalability. The development of COF-based theranostic materials faces challenges, such as scalability, reproducibility, and fine-tuning of properties for specific applications. Advances in synthetic methodologies, computational modeling, and understanding structure–property relationships drive the design of next-generation COF-based materials with enhanced functionalities for theranostics. The potential of COF-based materials in the biomedical field is in the starting stage and will open new doors in the future for exciting opportunities in advanced human healthcare.

## Abbreviations

MOFs: Metal–organic frameworks
COFs: Covalent–organic frameworks
ILs: Ionic liquids
CTFs: Covalent triazine-based frameworks
Azo: 4,4′-Azodianiline
Tp: 1,3,5-Triformylphloroglucinol
LAG: Liquid-assisted grinding
PTT: Photothermal therapy
PDT: Photodynamic therapy
TERM: Tissue engineering and regenerative medicine
DOX: Doxorubicin
5-FU: Fluorouracil
TTI-COF: Imine-based COF
TT-am: Triazine triphenyl amine
TT-ald: Triazine triphenyl aldehyde
IBU: Ibuprofen
APTES: 3-Aminopropyltriethoxysilane
PEG: Pegylated
CCM: Curcumin
PSs: Photosensitizers
BODIPY: Boron-dipyrromethene
ROS: Reactive oxygen species
PTA: Photothermal agent
NIR: Near infrared
TPAT: Tris(4-aminophenyl) amine
CAD: *cis*-Aconityl-DOX
TAPB: 1,3,5-Tris(4-aminophenyl) benzene
DMPT: 2,5-Dimethoxyterephthaldehyde
BD: Benzidine
